# NLRX1 Inhibits LPS-Induced Microglial Death via Inducing p62-Dependent HO-1 Expression, Inhibiting MLKL and Activating PARP-1

**DOI:** 10.3390/antiox13040481

**Published:** 2024-04-17

**Authors:** Yu-Ling Huang, Duen-Yi Huang, Vladlen Klochkov, Chi-Ming Chan, Yuan-Shen Chen, Wan-Wan Lin

**Affiliations:** 1 Department of Pharmacology, College of Medicine, National Taiwan University, Taipei 100233, Taiwan; 2 Department of Ophthalmology, Cardinal Tien Hospital, New Taipei City 23148, Taiwan; 3 School of Medicine, Fu Jen Catholic University, New Taipei City 242062, Taiwan; 4 Department of Neurosurgery, National Taiwan University, Yunlin Branch, Yunlin 640203, Taiwan; 5 Graduate Institute of Medical Sciences, Taipei Medical University, Taipei 110301, Taiwan

**Keywords:** NLRX1, microglia, HO-1, PARP-1, MLKL

## Abstract

The activation of microglia and the production of cytokines are key factors contributing to progressive neurodegeneration. Despite the well-recognized neuronal programmed cell death regulated by microglial activation, the death of microglia themselves is less investigated. Nucleotide-binding oligomerization domain, leucine-rich repeat-containing X1 (NLRX1) functions as a scaffolding protein and is involved in various central nervous system diseases. In this study, we used the SM826 microglial cells to understand the role of NLRX1 in lipopolysaccharide (LPS)-induced cell death. We found LPS-induced cell death is blocked by necrostatin-1 and zVAD. Meanwhile, LPS can activate poly (ADP-ribose) polymerase-1 (PARP-1) to reduce DNA damage and induce heme oxygenase (HO)-1 expression to counteract cell death. NLRX1 silencing and PARP-1 inhibition by olaparib enhance LPS-induced SM826 microglial cell death in an additive manner. Less PARylation and higher DNA damage are observed in NLRX1-silencing cells. Moreover, LPS-induced HO-1 gene and protein expression through the p62-Keap1-Nrf2 axis are attenuated by NLRX1 silencing. In addition, the Nrf2-mediated positive feedback regulation of p62 is accordingly reduced by NLRX1 silencing. Of note, NLRX1 silencing does not affect LPS-induced cellular reactive oxygen species (ROS) production but increases mixed lineage kinase domain-like pseudokinase (MLKL) activation and cell necroptosis. In addition, NLRX1 silencing blocks bafilomycin A1-induced PARP-1 activation. Taken together, for the first time, we demonstrate the role of NLRX1 in protecting microglia from LPS-induced cell death. The underlying protective mechanisms of NLRX1 include upregulating LPS-induced HO-1 expression via Nrf2-dependent p62 expression and downstream Keap1-Nrf2 axis, mediating PARP-1 activation for DNA repair via ROS- and autophagy-independent pathway, and reducing MLKL activation.

## 1. Introduction

Microglia are the resident macrophages of the CNS, and they perform functions to support neurons and surveil for pathogens. Once activated by stimuli, microglia proliferate, increase phagocytosis and release proinflammatory cytokines to resolve infection or tissue damage. Nevertheless, microglia also play important roles in neuroinflammation and neuronal cell death that are associated with the release of cytokines and production of ROS, and they contribute to many diseases of the brain [1,2,3,4]. In addition, microglial death during Alzheimer’s disease [5], Parkinson’s disease [6], and multiple sclerosis [7] helps fuel a neurotoxic environment. Compared to the well-recognized neuronal programmed cell death regulated by microglial activation, there still remains a gap in knowledge regarding the cell death mechanisms of microglia. TLR4 activation by LPS has been shown to induce microglial death [8,9,10]. Compared to peripheral macrophages, microglia exhibit high susceptibility to LPS [11,12]. Different death modes, including apoptosis [13,14,15,16], necroptosis [17,18], parthanatos [19] and pyroptosis [9], have been shown in LPS-induced microglial cell death. All these findings suggest that TLR4 plays an essential role in the pathogenesis of neurodegeneration.

NLRX1 is the only NOD-like receptor (NLR) to be targeted toward the mitochondria [20,21]. NLRX1 is ubiquitously expressed in different tissues of mammalian cells with cell type-specific differences in function [22]. The major role of NLRX1 in immunity is its involvement in negative regulation of mitochondrial antiviral-signaling protein (MAVS)-mediated type I interferon (IFN) signaling by directly interacting with MAVS through its LRR domain [23]. NLRX1 can interact with TUFM, which subsequently associates with autophagic proteins to promote virus-induced autophagy. To date, the role of NLRX1 in cell death remains controversial and is cell type- and cellular context-dependent [24,25,26].

Recently, studies support the importance of NLRX1 in brain injury following aneurysm and neurodegenerative diseases [27,28]. Therefore, in this study, we were interested to explore the role of NLRX1 in LPS-induced microglial cell death and the underlying mechanisms. We covered PARP-1, HO-1 and necroptosis. The reasons to address them are because the PARP-1 inhibitor attenuates microglial activation and neurodegeneration [29,30,31,32]; HO-1 exerts the anti-inflammation and anti-apoptosis responses in LPS-stimulated microglia [15,33]; and necroptosis is induced by LPS in microglia [17,18]. However, to date, the roles of NLRX1 and PARP-1 in regulating microglial death are still poorly understood.

## 2. Materials and Methods

### 2.1. Reagents and Antibodies

Dulbecco’s modified Eagle’s medium (DMEM), fetal bovine serum (FBS) and trypsin EDTA were purchased from Gibco (Carlsbad, CA, USA). Penicillin–streptomycin antibiotic solution was obtained from Biological Industries (Kibbutz Beit-Haemek, Israel). Dulbecco’s phosphate buffered saline (PBS), sodium bicarbonate, puromycin, LPS, N-acetyl-L-cysteine (NAC), hemin and protease inhibitor cocktails were purchased from Sigma-Aldrich (St. Louis, MO, USA). Olaparib was obtained from Selleck Chemicals (Houston, TX, USA). Zinc protoporphyrin (ZnPP) was purchased from MedChemExpress (Monmouth Junction, NJ, USA). Antibodies of NLRX1 (sc-374514) and β-actin (sc-47778) were obtained from Santa Cruz Biotechnology (Dallas, TX, USA). Antibodies of γH2AX (#9718), PARP-1 (#9542), and p62 (#5114) were obtained from Cell Signaling Technology (Beverly, MA, USA). Antibodies of Nrf2 (GTX103322) and microtubule-associated protein 1A/1B-light chain 3 (LC3) (GTX127375) were obtained from GeneTex (Irvine, CA, USA). The antibody of HO-1 (ADI-SPA-896) was obtained from Enzo Life Science (Farmingdale, NY, USA). The antibody of poly(ADP-ribose) (PAR) (4335-MC-100) was obtained from R&D Systems (Minneapolis, MN, USA).

### 2.2. Cell Culture

The murine SM826 microglial cell line was gifted by Dr. Feng-Shiun Shie (Center for Neuropsychiatric Research, National Health Research Institutes, Taiwan), which was spontaneously immortalized and developed from primary microglial cultures using murine neonates with a BALB/c background [34]. Cells were cultured in complete high-glucose DMEM containing 10% FBS and 1% penicillin–streptomycin.

### 2.3. Generation of shRNA and siRNA Knockdown Cell Lines

When generating stable short hairpin RNA (shRNA) knockdown cell lines, lentivirus particles encoding shRNA targeting NLRX1 were used for transfection. A density of 3 × 10^5^ cells per well was seeded in 6-well plates and maintained overnight for attachment. After removing the supernatant, a mixture of polybrene (8 μg/mL) and media without penicillin–streptomycin was added to the wells and then treated with lentivirus. After 24 h of infection, the lentivirus-containing supernatants were removed and fresh growth media were added to the wells with puromycin (3 μg/mL). The media containing puromycin were changed every 3 to 4 days during the selection. Transfected cells were selected with puromycin for 2 weeks to select successful transfection. Finally, the stable knockdown cell lines were kept with puromycin (1 μg/mL) for every subculture.

Mouse sip62 and scramble nonspecific siRNA were obtained from Sigma-Aldrich (St. Louis, MO, USA) and Santa Cruz Biotechnology (Santa Cruz, CA, USA). Wild-type SM826 cells were transfected with 800 nM siRNA by DharmaFECT Transfection Reagents (Dharmacon Research, Lafayette, CO, USA). After 24 h, the medium was changed to complete medium, and after another 48 h, the cells were treated with the indicated drugs and then harvested for analysis.

### 2.4. Annexin V/PI Staining

Cells were seeded at 2 × 10^5^ cells per well in 6-well plates and incubated overnight at 37 °C, followed by the treatment as mentioned. Following the treatment, the cells were washed with PBS, collected with trypsin in 1.5 mL Eppendorf tubes and centrifuged at 2000 rpm, 4 °C, for 5 min. The lysates were suspended and stained with an FITC Annexin V Apoptosis Kit (BioLegend, San Diego, CA, USA). Annexin V-FITC and propidium iodide (PI) diluted with Annexin V binding buffer were used to stain the samples at room temperature for 15–30 min. After staining, the samples were transferred from Eppendorf to flow tubes and then 400 μL Annexin V binding buffer was added. Cell viability was evaluated with a flow cytometer (BD FACSCalibur, Franklin Lakes, NJ, USA) available for FL1 (Annexin V) and FL2 (PI) bivariate analyses. Analysis by calculating the percentage of the cells in the respective quadrants was performed using CellQuest PRO software 4.0.1.

### 2.5. Measurement of Intracellular ROS Production

The intracellular levels of hydrogen peroxide (H_2_O_2_) were measured with dichlorodihydrofluorescein diacetate (DCFDA). Cells were seeded in 6-well plates overnight, followed by the indicated treatments. At the endpoint, the cells were washed with PBS, collected with trypsin in 1.5 mL Eppendorf tubes and centrifuged at 2000 rpm, 4 °C, for 5 min. The lysates were suspended in 500 μL PBS with DCFDA (10 μM) at 37 °C for 30 min. The cells were then washed with PBS and collected as single-cell suspensions. Each flow tube contained 500 μL PBS and was evaluated with a flow cytometer (BD FACSCalibur, Franklin Lakes, NJ, USA) available for FL1 by fluorescence signal under excitation and emission at 488 nm and 520 nm, respectively.

### 2.6. Immunoblotting

After drug treatment and aspirating the culture media, the cells were washed with PBS and then lysed with RIPA lysis buffer, which contained 50 mM Tris-HCl pH 7.6, 150 mM NaCl, 1% Triton X-100, 0.1% sodium dodecyl sulfate (SDS), 0.1% deoxycholate, 2 mM NaF, 2 mM Na_3_VO_4_, 1 mM phenylmethylsulphonyl fluoride and protease inhibitor cocktails. After harvest, the cell lysates were sonicated for 5 to 10 sec for complete the cell lysis and centrifuged at 13,000 rpm, 4 °C, for 15 min. The supernatants were collected to determine the protein concentration by Bio-Rad protein assay, and the samples were heated at 98 °C for 5 min. About 20 μg of soluble protein was subjected to 8~15% SDS-polyacrylamide gel electrophoresis, and transferred to Immobilon-P. The 5% nonfat milk in Tris-buffer saline with Tween 20 (TBST) was used for blocking the nonspecific binding for 1 h at room temperature. After incubating with the primary antibodies with gentle shaking overnight at 4 °C, the membranes were washed with TBST three times and incubated with horseradish-peroxidase-linked secondary antibodies with shaking for 1 h at room temperature. After washing with TBST three times, the protein bands were detected on a Bio-Rad UVP system with ECL reagents. β-actin was used as the internal control to ensure an equal amount of sample loading. The Western blotting images were quantified with ImageLab 6.1 software.

### 2.7. Reverse-Transcription and Real-Time Polymerase Chain Reaction (RT-PCR)

The mRNA expressions of NLRX1, HO-1, Keap1, Nrf2, p62, LC3 and β-actin were determined by real-time PCR analysis. The specific primers for these genes were shown in Supplementary Table S1. After drug treatment and aspirating the culture media, the cells were washed with PBS and then lysed with TRIzol reagents (Invitrogen, Waltham, MA, USA) and the RNAs were extracted. Next, 1 μg of total RNA underwent reverse-transcription and conversion to cDNA. SYBR Green Master Mix was applied for real-time PCR in 96-well plates, in which each well contained a 25 μL mixture of diluted cDNA, a set of gene-specific primers and Master Mix. The PCR samples were analyzed by a QuantStudio 5 system (Applied Biosystems, Oakland, CA, USA). All the mRNA levels of the target genes were normalized with β-actin and represented as the fold changes compared to untreated cells.

### 2.8. Confocal Microscopy

After treatment, SM826 microglial cells were fixed with 4% paraformaldehyde and permeabilized with 0.2% Triton X-100 in PBS for 20 min. The samples were blocked with 4% BSA for 1 h and incubated with primary antibody for 2 h at room temperature or overnight at 4 °C after aspiration of the blocking solution. The primary antibody was then discarded and the cells were washed three times with PBS. Afterwards, the samples were incubated with fluorochrome-conjugated secondary antibody for 1 h in the dark. Following immunostaining process, the coverslip was counterstained with 4′,6-diamidino-2-phenylindole (DAPI) and mounted on microscope slides in the dark. The samples were analyzed with an LSM 880 confocal microscope (Zeiss, Oberkochen, Germany).

### 2.9. Quantification and Statistical Analysis

Data were presented as the mean ± S.E.M. Student’s *t* test was used to assess the statistical significance of the differences between the samples, and *p* < 0.05 was considered statistically significant.

## 3. Results

### 3.1. NLRX1 Silencing Enhances LPS-Induced Mixed-Type Cell Death in SM826 Cells, Which Is Additive to the PARP-1 Inhibitor

**Figure 1:**
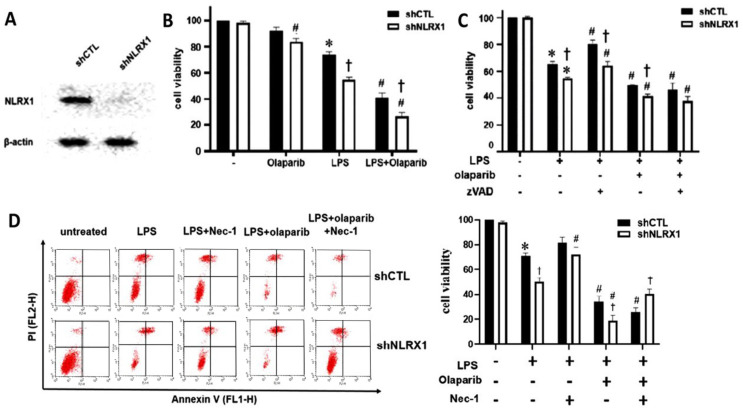
NLRX1 silencing increases SM826 cell death upon LPS/olaparib stimulation. (**A**) Establishment of NLRX1–silencing SM826 microglia. (**B**–**D**) Cell viability in shCTL and shNLRX1 SM826 cells treated with LPS (100 ng/mL), olaparib (10 μM) (**B**–**D**), zVAD (20 μM) (**C**) and/or necrostatin−1 (10 μM) (**D**) for 24 h was determined by Annexin V/PI staining. The quantification data were presented as the mean ± S.E.M from 3–5 independent experiments. *, *p* < 0.05 indicating a significant effect of LPS compared to the untreated group. #, *p* < 0.05 indicating significant effects of olaparib, zVAD and necrostatin−1 to change control LPS response, respectively, in the shCTL or shCASK group. †, *p* < 0.05 indicating a significant difference in the LPS response between the shCTL and shNLRX1 groups.

After confirming the effective silencing of the NLRX1 protein (Figure 1A), LPS (100 ng/mL) and/or olaparib (10 μM) were treated for 24 h. We found olaparib enhanced cell death in response to LPS, and NLRX1 silencing further increased the death susceptibility to LPS, olaparib and LPS/olaparib (Figure 1B). In addition, zVAD only partially reversed cell death, and NLRX1 silencing still increased cell death under zVAD treatment (Figure 1C). These findings suggest that LPS induces a mixed-type cell death in microglia, and PARP-1 and NLRX1 can protect cells via a caspase-independent mechanism.

After suggesting the existence of a caspase-independent death mechanism in LPS-treated SM826 cells, we considered necroptosis, which can be caused by LPS [35,36]. Necroptosis is mediated by the receptor-interacting serine/threonine-protein kinase 1 (RIP1) kinase activity-dependent formation of the RIP1–RIP3–MLKL complex. We found RIP1 inhibitor necrostatin-1 (10 μM) reversed LPS-induced cell death in shNLRX1 cells but not in shCTL cells. Such cell protection was also observed in LPS/olaparib-treated shNLRX1 cells (Figure 1D). We suggest that NLRX1 might be able to negatively regulate necroptosis signaling after LPS stimulation.

### 3.2. NLRX1 Silencing Inhibits LPS-Induced PARP-1 Activation and DNA Repair

**Figure 2:**
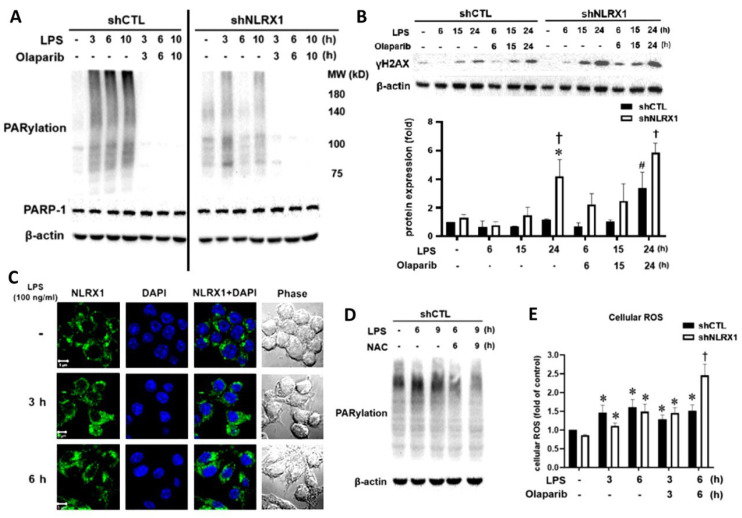
NLRX1 silencing inhibits PARP−1 activity. Cells were treated with LPS (100 ng/mL), olaparib (10 μM) and/or NAC (5 mM) for the indicated times. (**A**,**B**,**D**) Total cell lysates were analyzed by immunoblotting to determine PARylation, PAPR−1 (**A**,**D**) and γH2AX (**B**). (**C**) Confocal images show the subcellular localization of NLRX1. Scale bar: 5 μm. (**E**) Cellular ROS production was determined by DCFDA staining and flow cytometry. The quantification data were presented as the mean ± S.E.M from 3–4 independent experiments. *, *p* < 0.05 indicates a significant effect of LPS compared to the untreated group. #, *p* < 0.05 indicates significant effects of olaparib in changing the LPS response. †, *p* < 0.05 indicates a significant difference in the LPS response between the shCTL and shNLRX1 groups.

LPS has been shown to activate PARP-1 in macrophages/microglia to induce parthanatos [19]. Here, we sought to understand if NLRX1 plays a role in the PARP-1-mediated DNA repair process. We found that LPS induced PARylation (the index of PARP-1 activation) within 3–10 h of treatment, and this effect was abolished by olaparib. Of note, LPS-induced PARylation was attenuated by shNLRX1 (Figure 2A). Along with LPS-induced PARP-1 activation, γH2AX expression (the index of DNA damage) was induced, and this effect was enhanced by olaparib and shNLRX1 (Figure 2B), suggesting NLRX1 is involved in LPS-induced PARP-1 activation for DNA repair, and this action contributes to cell protection. To explore if NLRX1 might directly affect PARP-1 activation, we conducted confocal microscopy. The data revealed that NLRX1 is not located in the nucleus (Figure 2C). Apart from DNA damage, oxidative stress triggers PARP-1 activation [37,38,39,40,41]. Therefore, we evaluated the role of ROS in LPS-induced PARylation. We found that the increase in PARP-1 activity under LPS stimulation was blocked by ROS scavenger NAC (Figure 2D). Moreover, LPS induced a moderate increase in ROS production and this effect was not affected by NLRX1 silencing or olaparib. However, under olaparib treatment, the LPS-induced ROS production at 6 h was significantly enhanced by shNLRX1 (Figure 2E), correlating with higher DNA damage (Figure 2B) and cell death (Figure 1B) in LPS/olaparib-treated shNLRX1 cells. These data suggest that NLRX1 not only involves in PARP-1 activation independent of ROS but also exerts a PARP-1-independent cell protective mechanism.

### 3.3. NLRX1 Silencing Enhances MLKL Activation

**Figure 3:**
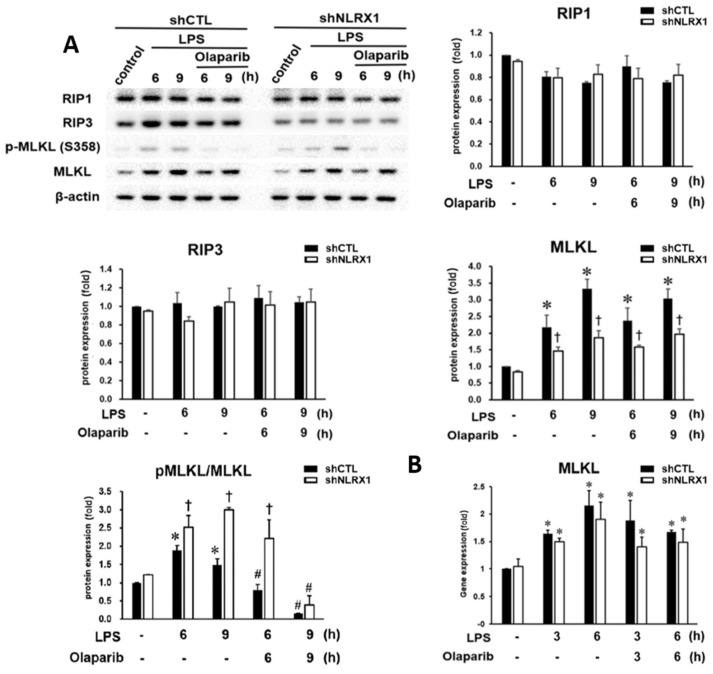
NLRX1 negatively regulates LPS–induced MLKL activation. shCTL and shNLRX1 cells were treated with LPS (100 ng/mL) and/or olaparib (10 μM) for the indicated times. (**A**) Total cell lysates were analyzed by immunoblotting to determine RIP1, RIP3, MLKL and MLKL–p. (**B**) Real–time PCR was used to determine MLKL. The data were presented as the mean ± S.E.M. from 3–5 independent experiments. *, *p* < 0.05 indicates the significant effect of LPS compared to the respective untreated shCTL or shNLRX1 group. #, *p* < 0.05 indicates significant effects of olaparib compared to the respective LPS–treated shCTL or shNLRX1 group. †, *p* < 0.05 indicates a significant difference between the shCTL and shNLRX1 groups.

When studying the necroptosis-related proteins RIP1, RIP3 and MLKL, our data unexpectedly revealed the ability of LPS to increase MLKL protein expression and MLKL activation as indexed by the increased pMLKL/MLKL ratio, without affecting the RIP1 and RIP3 protein levels (Figure 3A). Accordingly, LPS upregulated MLKL gene expression (Figure 3B). Although shNLRX1 decreased LPS-induced MLKL protein expression, the increased MLKL gene expression was not changed by shNLRX1 (Figure 3B). It is also interesting to note that the pMLKL/MLKL ratio was enhanced by shNLRX1 (Figure 3A). Although olaparib did not affect MLKL gene and protein expression, it reduced the pMLKL/MLKL ratio under LPS stimulation in both shCTL and shNLRX1 cells (Figure 3A). These results suggest that PARP-1 activity is involved in LPS-induced MLKL activation, but necroptosis does not contribute to olaparib-induced enhancement of cell death, as our data shown in Figure 1D. In addition, NLRX1 performs negative regulation in MLKL activation and prevents microglial necroptosis upon LPS stimulation.

### 3.4. NLRX1 Silencing Reduces LPS-Induced HO-1 Expression

**Figure 4:**
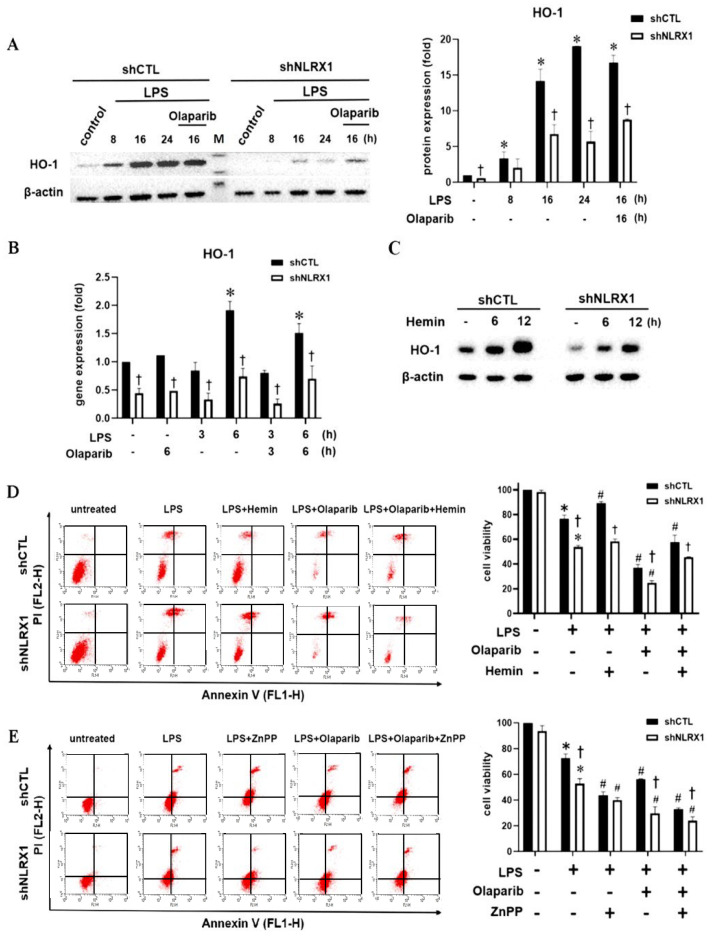
LPS–induced protective HO−1 expression is attenuated by NLRX1 silencing. shCTL and shNLRX1 SM826 cells were treated with LPS (100 ng/mL), olaparib (10 μM), hemin (20 μM) and/or ZnPP (10 μM) for the indicated times. (**A**,**C**) Total cell lysates were analyzed by immunoblotting to determine HO−1. (**B**) Quantitative real–time PCR was used to determine the gene expression of HO−1. (**D**,**E**) After drug treatment for 24 h, cell viability was determined. The data were presented as the mean ± S.E.M from 3 independent experiments. *, *p* < 0.05 indicates a significant effect of LPS (100 ng/mL) compared to the untreated shCTL group. #, *p* < 0.05 indicates the significant effects of olaparib, hemin and ZnPP on LPS–induced cell death. †, *p* < 0.05 indicates a significant difference in the LPS response between the shCTL and shNLRX1 groups. In (**A**), it is the marker indicated as “M” in lane 6.

In the CNS, HO-1 is mainly induced in microglia by oxidative stimulus and is necessary to attenuate neuronal cell death and maintain cognitive functions [42]. Because LPS has been shown to upregulate HO-1 for cell protection in microglia [15,43], we checked if HO-1 might be involved in NLRX1 regulation of cell viability. We first confirmed that LPS induced HO-1 protein expression in SM826 microglial cells, and this effect was unaffected by olaparib but was attenuated by NLRX1 silencing (Figure 4A). Accordingly, NLRX1 silencing significantly reduced the basal and LPS-induced HO-1 gene expression, and this effect was not changed by olaparib (Figure 4B). Next, we treated microglia with hemin (20 μM), the inducer of HO-1, and found that the HO-1 induction was also attenuated by NLRX1 silencing (Figure 4C). Under this condition, hemin reduced LPS-induced death in shCTL cells but not in shNLRX1 cells. On the other hand, the enhancement of cell death caused by olaparib was partially protected by hemin in both shCTL and shNLRX1 cells (Figure 4D). In contrast, we found the HO-1 inhibitor ZnPP increased LPS- and/or olaparib-induced cell death, especially in shCTL cells (Figure 4E). We suggest that HO-1 induction counteracts LPS/olaparib-induced microglial death, and the attenuation of HO-1 expression by shNLRX1 leads to enhanced cell death.

### 3.5. NLRX1 Silencing Decreases the LPS-Induced Nrf2/HO-1 Pathway via Inhibiting Keap1 Downregulation

**Figure 5:**
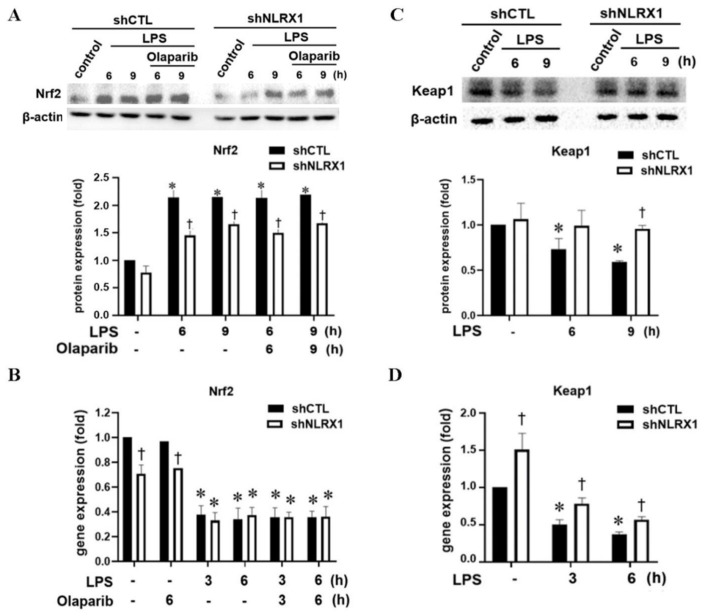
NLRX1 silencing decreases the LPS–induced Keap1/Nrf2/HO−1 pathway. Here, shCTL and shNLRX1 SM826 cells were treated with LPS (100 ng/mL) and/or olaparib (10 μM) for the indicated times. (**A**,**C**) Total cell lysates were analyzed by immunoblotting to determine Nrf2 and Keap1. (**B**,**D**) Real–time PCR was used to determine the gene expression of Nrf2 and Keap1. The data were presented as the mean ± S.E.M. from 3–4 independent experiments. *, *p* < 0.05 indicates a significant effect of LPS (100 ng/mL) compared to the untreated respective shCTL or shNLRX1 group. †, *p* < 0.05 indicates a significant difference between the shCTL and shNLRX1 groups.

The major redox-sensitive transcription factor that binds to the HO-1 gene is Nrf2, and it becomes a potential therapeutic target for numerous disorders [44,45,46]. Therefore, we sought to understand if Keap1-Nrf2 is accordingly regulated by NLRX1. As expected, Nrf2 protein expression was increased under LPS stimulation, and this effect was unaffected by olaparib but was attenuated by NLRX1 silencing (Figure 5A). In contrast, LPS inhibited the mRNA level of Nrf2, and this effect was not altered by NLRX1 silencing, despite the moderate inhibition of Nrf2 gene expression at the resting state by NLRX1 silencing (Figure 5B). These results reveal that LPS induces Nrf2 protein expression independent of gene transcription, and NLRX1 might be involved in the post-transcriptional regulation of Nrf2. As to Keap1, our immunoblotting data revealed the ability of LPS to downregulate the Keap1 protein and gene expression in shCTL cells (Figure 5C,D). It is worth noting that the LPS-induced downregulation of the Keap1 protein was blocked by shNLRX1 (Figure 5C). These findings explain why NLRX1 silencing can reduce LPS-induced Nrf2 protein expression. Of note, although NLRX1 silencing significantly increased Keap1 gene expression at the resting state, it did not alter the inhibitory effect of LPS on Keap1 gene expression (Figure 5D). We suggest that NLRX1 is a positive regulator of the Nrf2/HO-1 pathway, possibly via de-stabilization of the Keap1 protein and prevention of Nrf2 from performing ubiquitination and degradation.

### 3.6. NLRX1 Silencing Suppresses LPS-Induced Autophagy and p62 Upregulation

**Figure 6:**
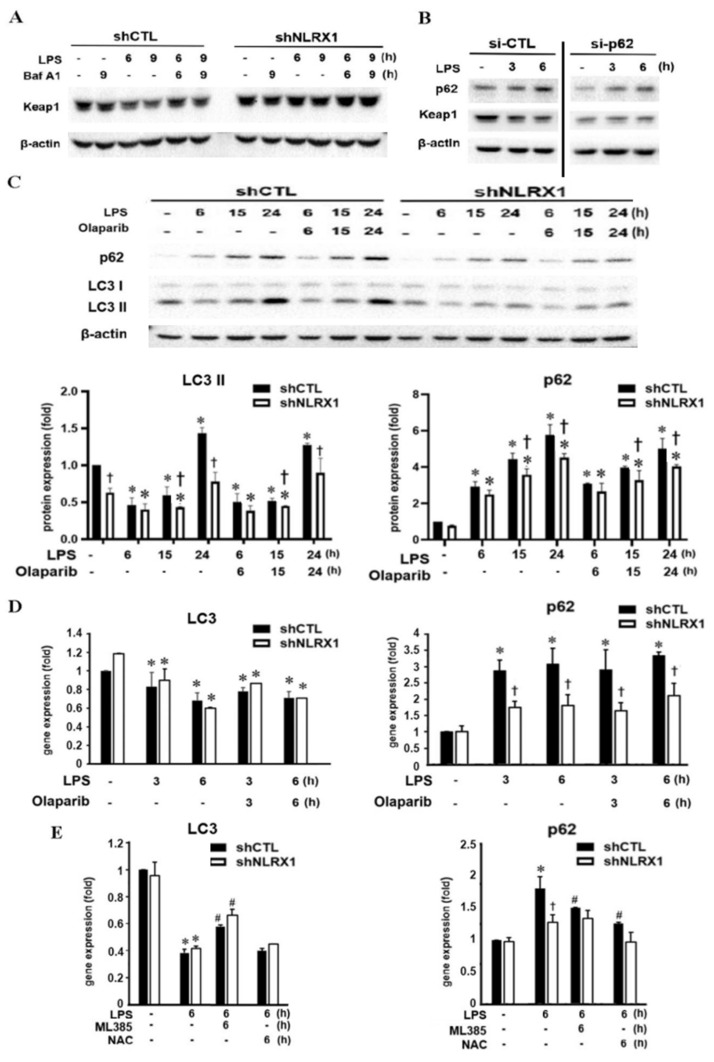
NLRX1 silencing inhibits LPS–induced p62 and LC3II expression. Here, shCTL and shNLRX1 cells were treated with LPS (100 ng/mL), olaparib (10 μM), bafilomycin A1 (100 nM), ML385 (10 μM) and/or NAC (5 mM). (**A**–**C**) Total cell lysates were analyzed by immunoblotting. (**D**,**E**) Real–time PCR was used to determine p62 and LC3 gene expression. The data were presented as the mean ± S.E.M. from 3–4 independent experiments. *, *p* < 0.05 indicates a significant effect of LPS (100 ng/mL) compared to the untreated respective shCTL and shNLRX1 groups. #, *p* < 0.05 indicates the significant effects of ML385 and NAC on the LPS–induced responses to LC3 or p62 gene expression compared to the respective shCTL and shNLRX1 groups without drug treatment. †, *p* < 0.05 indicates a significant difference between the shCTL and shNLRX1 groups.

Previously, the classical autophagy adaptor molecule p62 has been shown to induce the Keap1-HO-1 pathway by direct interaction with Keap1 for degradation [47,48,49,50]. Vice versa, the dysfunction of autophagy results in prolonged Nrf2 activation in a p62-dependent manner [51]. Therefore, we sought to investigate the role of autophagy in LPS-induced microglial death and its relationship to NLRX1. First, we found that the autophagy inhibitor bafilomycin A1 (Baf A1) inhibited the Keap1 downregulation caused by LPS in shCTL cells. In shNLRX1 cells, the Keap1 protein remained at its increased level after Baf A1 treatment (Figure 6A). These findings support an autophagy-dependent mechanism for the Nrf2/HO-1 axis upon LPS stimulation. To further understand if p62 is involved in LPS-induced Keap1 downregulation, we measured the p62 expression and determined the effect of p62 silencing. LPS time-dependently upregulated p62 protein expression, which was accompanied by the downregulation of Keap1. In the p62-silencing condition, LPS no longer downregulated Keap1 (Figure 6B). These findings confirm the action of LPS in the activation of the p62/Keap1/Nrf2/HO-1 axis.

Next, we checked if autophagy is involved in the action of LPS and how NLRX1 regulates p62 expression. Because PARP-1 is involved in inhibiting autophagy by either the induction of PARylation in autophagy-related proteins or the inhibition of SIRT1 [52,53], or in inducing autophagy by activating AMPK [54], we also investigated this possibility in LPS-stimulated microglia. When examining the LC3II level as the index of autophagy, we found LPS caused a biphasic change. The LC3II protein level was decreased after LPS stimulation for 6 h, then recovered after 15 h and further increased at 24 h. This biphasic change was not affected by olaparib. However, in shNLRX1 cells, the basal protein level of LC3II was lower than that in control cells, and the LPS-induced increase in LC3II expression at the late phase was diminished (Figure 6C). Moreover, LPS time-dependently induced p62 protein expression within 24 h, and this response was unaffected by olaparib but was inhibited by NLRX1 silencing (Figure 6C). Further analyzing the gene transcription of LC3 and p62, we found LPS slightly decreased the LC3 mRNA level but markedly increased the p62 mRNA level within 6 h. Olaparib did not change LC3 and p62 gene expression, while NLRX1 silencing inhibited LPS-induced p62 gene expression without affecting the effect of LPS on the downregulation of LC3 gene expression (Figure 6D). These findings highlight a new function of NLRX1 in upregulating HO-1 via the autophagic induction of p62, leading to the downregulation of Keap1 and upregulation of Nrf2.

It was reported that the LPS-induced p62 gene upregulation in macrophages is caused by Nrf2 activation [55]. Here, we found the Nrf2 inhibitor ML385 reversed LC3 and p62 expression in response to LPS in shCTL cells (Figure 6E). Moreover, when determining the role of oxidative stress in p62 and LC3 responses, we found the antioxidant NAC only reversed the induction effect of LPS on p62 gene expression but did not alter the LPS-induced suppression of LC3 gene expression in shCTL cells (Figure 6E). Overall, we suggest that NLRX1 is involved in LPS-induced p62 expression and autophagy. NLRX1 plays a role in p62 gene expression, which is mediated by the Nrf2 and ROS pathways. NLRX1 also mediates LPS-induced LC3II expression without affecting LPS-induced suppression of LC3 gene expression, which is mediated by Nrf2.

### 3.7. NLRX1 Silencing Inhibits PARP-1 Activation Independently of Autophagy Suppression

**Figure 7:**
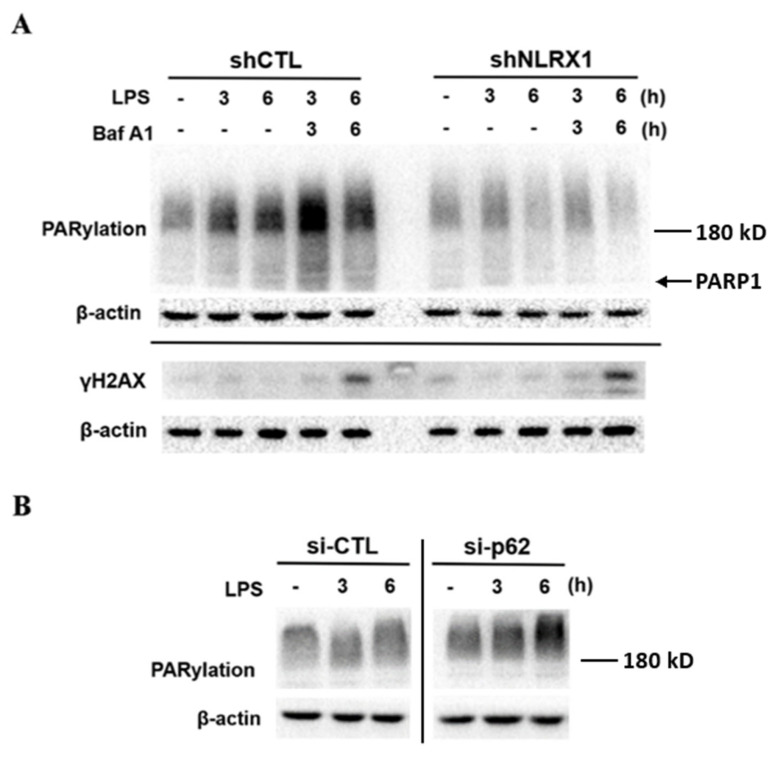
Autophagy inhibits PARP−1 activity, but inhibition of PARP−1 activation by NLRX1 silencing might be independent of autophagy suppression. (**A**) The shCTL and shNLRX1 cells were treated with LPS (100 ng/mL) and/or bafilomycin A1 (100 nM). (**B**) The shCTL cells were pretreated with p62 siRNA to knockdown p62 followed by LPS stimulation. Total cell lysates were analyzed by immunoblotting to determine PARylation and γH2AX.

We observed that NLRX1 silencing reduces LPS-induced PARP-1 activation (Figure 2A) independently of ROS production (Figure 2E). The deficiency of autophagy has been linked to an increase in genomic instability [56]. We were also interested in understanding if attenuating autophagy by NLRX1 silencing might induce genomic instability and PARP1-1 activation. Our data revealed that the autophagy inhibitor bafilomycin A1 indeed enhances LPS-induced PARylation and the DNA damage index γH2AX (Figure 7A). Meanwhile, p62 silencing also increased LPS-induced PARP-1 activation (Figure 7B). These findings indicate autophagy can regulate the DNA damage associated PARP-1 activity. Of note, bafilomycin A1 did not increase PARylation in LPS-treated shNLRX1 cells where PARP-1 activity had been suppressed (Figure 7A). Given that NLRX1 silencing suppresses LC3II and p62 protein expression under LPS stimulation (Figure 6C), we suggest that NLRX1 participates in PARP-1 activation and the DNA repair might be independent of autophagy.

## 4. Discussion

NLRX1 has been reported to be associated with several diseases [20,57,58,59,60]. Decreased NLRX1 expression was observed in human intracranial aneurysms and in hypoxic cardiomyocytes, kidney, brain and intestine [27,61,62,63,64]. Decreased NLRX1 expression in chronic obstructive pulmonary disease patients is linked to pulmonary disease severity and poor prognosis [65]. According to our findings, NLRX1 silencing enhances LPS-induced SM826 cell death, supporting the protective role of NLRX1 in microglia. In this study, for the first time, we demonstrate the crucial role of NLRX1 in protecting LPS-induced mixed-type cell death in the SM826 microglial cells. There are several underlying mechanisms for such protection, including inducing the p62/Keap1/Nrf2/HO-1 axis via autophagy, inhibiting MLKL activity and enhancing PARP-1-dependent DNA repair.

TLR4-mediated autophagy plays an important role in bacterial clearance. In RAW264.7 macrophages, LPS induces p62 but not LC3 expression, and in primary microglia, LPS decreases LC3 gene expression [66,67]. In this study, we observed the ability of LPS to initially downregulate LC3 protein expression via gene transcription in SM826 cells (Figure 6C,D), and this effect is followed by the protein upregulation (Figure 6C). However, NLRX1 silencing only attenuates the LPS response in LC3 protein expression but not in LC3 gene expression. Here, we also observed that the LPS-induced downregulation of LC3 mRNA expression is partially reversed by the Nrf2 inhibitor (Figure 6E). These findings are consistent with the previous notion that Nrf2 can upregulate LC3 gene expression [68,69]. Likewise, p62 is a key regulator required for autophagy, and our data reveal its time-dependent upregulation by LPS at the transcriptional level (Figure 6C,D). Consistent with previous studies in macrophages [55,69], LPS-induced p62 gene upregulation in microglia is also caused by ROS and Nrf2 activation (Figure 6E). In addition, our data suggest NLRX1 might regulate this effect of LPS via the Nrf2 pathway. Because shNLRX1 does not change LPS-induced ROS production (Figure 2E), we exclude the role of ROS in the action mediated by NLRX1.

PARP-1 activation in response to DNA damage and oxidative stress mediates a variety of cellular responses, including cell death. It was reported that PARP-1 plays dual roles in modulating cell death. Under the condition of moderate DNA damage, PARP-1 has a protective role. On the other hand, after excessive DNA damage or in inflammatory pathological situations, PARP-1 plays a crucial role in cell death and neurodegeneration by its over-activation, leading to ATP depletion [70]. Similarly, the induced PARP-1 activation, PAR formation and AIF translocation contribute to the LPS-induced parthanatos in macrophages [19]. However, the role of PARP-1 in LPS-stimulated microglia is different from its deteriorated role in neurons and macrophages under stress. Our data reveal the ability of the PARP-1 inhibitor olaparib to increase cell death in LPS-stimulated microglia, suggesting DNA repair by PARP-1 exerts a protective effect. In this study, we also demonstrated that NLRX1 mediates cell protection via PARP-1-dependent and -independent mechanisms. The former is based on the observation of less PARylation (Figure 2A) and higher DNA damage in shNLRX1 cells (Figure 2B), mimicking the effect of olaparib. The latter reveals that NLRX1 action beyond the regulation of PARP-1 is based on the additive effect of olaparib and shNLRX1 on cell fate control (Figure 1B).

As to the role of NLRX1 in regulating LPS-induced PARP-1 activation, we suggest it results from an ROS-independent mechanism. We found LPS-induced ROS production is not affected by NLRX1 silencing (Figure 2E). Because NLRX1 is not localized in the nuclei and does not enter into the nuclei upon LPS stimulation (Figure 2C), the possible direct interaction between NLRX1 and PARP-1 is excluded. Our co-immunoprecipitation data further rule out the direct interaction between NLRX1 and PARP-1. Another possible mechanism for regulating PARP-1 is via autophagy. This notion is based on the finding that autophagy deficiency increases genomic instability and subsequent PARP-1 activation [56]. LC3 and ULK1 knockout were shown to increase the accumulated micronuclei, a diagnostic marker of genomic instability. Other studies also highlight the importance of autophagy in the DNA repair process [71,72]. To address if autophagy might regulate PARP-1 activation in our study, we tested the effects of the autophagy inhibitor bafilomycin A1 and p62 silencing. Our data revealed that bafilomycin A1 indeed enhances LPS-induced PARylation and the DNA damage index (Figure 7A). Meanwhile, p62 silencing also increases LPS-induced PARP-1 activation (Figure 7B). These findings suggest autophagy is involved in downregulating PARP-1 activity. Of note, bafilomycin A1 does not increase PARylation in LPS-treated shNLRX1 cells. Because NLRX1 silencing suppresses LC3II and p62 expression under LPS stimulation (Figure 6), we suggest that NLRX1 participates in PARP-1 activation and DNA repair might be independent of autophagy. At this moment, it is still unclear how NLRX1 regulates PARP-1. On the other hand, because hemin can still protect LPS-induced cell death under olaparib treatment (Figure 4D), we rule out the involvement of PARP-1 in the cell protective function of HO-1. Due to the different subcellular localizations of NLRX1 and PARP-1, we suggest an indirect action regulated by NLRX1 in the mitochondria to enhance PARP-1 activation. We still need to clarify this unidentified mechanism between NLRX1 and PARP-1 in the future.

The Keap1/Nrf2/HO-1 axis is thought to be cytoprotective in microglia by reducing oxidative stress and inflammatory responses [73]. Keap1 is a component of the CUL3-based E3 ubiquitin ligase complex and controls the stability of Nrf2 by targeting it for ubiquitination and proteasomal degradation. When exposed to oxidative stress, the reactive cysteine residues of Keap1 are directly modified, which reduces the ubiquitin E3 ligase activity of the Keap1–CUL3 complex and results in Nrf2 protein stabilization [48,49,50,51]. In our findings, LPS-induced the downregulation of the Keap1 gene and protein expression was reversed by NLRX1 silencing (Figure 5C,D), and consequently, the inductions of the downstream proteins Nrf2 and HO-1 were attenuated (Figure 4A and Figure 5A). Although the ROS scavenger NAC is able to counteract this phenomenon (Figure 6E), ROS induction by LPS is not affected in shNLRX1 cells (Figure 2E), indicating the regulation of the Keap1/Nrf2/HO-1 pathway by NLRX1 is ROS-independent. Regarding the regulation of Keap1 mRNA expression, one study has shown the possible transcription factors that bind to the Keap1 promoter, including BRD4, SP1 and AP2. On the contrary, Sirt6 deacetylase decreases Keap1 transcription [74]. The potential mechanism of NLRX1 in modulating Keap1 gene expression needs further investigation. In addition to the transcriptional regulation, we also confirmed that the stability of Keap1 is affected by NLRX1. In shNLRX1 cells, autophagy is downregulated under LPS stimulation, and treatment of the autophagy inhibitor bafilomycin A1 and p62 silencing could reverse LPS-induced Keap1 downregulation (Figure 6A,B). Indeed, studies have shown that LPS-induced Keap1 protein reduction occurs through autophagy but not proteasome degradation in RAW 264.7 macrophages [75]. Of note, p62 is a direct mediator to interaction with Keap1, and the knockdown of p62 prevents LPS-induced Keap1 degradation. Also, NLRX1 was reported to be indispensable for autophagy induction. The NLRX1-interacting partner, TUFM, interacts with Atg5-Atg12 and Atg16L1, and it promotes autophagy [76,77]. Our data also support NLRX1 as a positive regulator of autophagy, and NLRX1 mediates Keap1 protein downregulation by autophagy. Surprisingly, we observed that LPS is capable of reducing Nrf2 gene expression (Figure 5B) while increasing its protein expression (Figure 5A). This effect was also found in LPS-treated macrophages, but the underlying mechanisms remain unknown [78].

Necroptosis is associated with the pathogenesis of Alzheimer’s disease, Parkinson’s disease, and traumatic brain injury [10,17,18,35]. In this study, we observed that LPS upregulates MLKL protein and gene expression (Figure 3A,B), and it increases the pMLKL/MLKL ratio without affecting the RIP1 and RIP3 protein levels (Figure 3A). Unexpectedly, NLRX1 silencing enhances the pMLKL/MLKL ratio while decreasing MLKL protein expression under LPS treatment (Figure 3A). For MLKL gene expression, IFNγ upregulates MLKL mRNA in breast carcinoma and cervical carcinoma cells, and IRF1 or STAT1 knockout reverses IFNγ-mediated induction of the MLKL mRNA level [79]. Because we did not observe the inhibition of MLKL gene expression in LPS-stimulated shNLRX1 cells, we exclude the contribution of IFNβ to MLKL gene expression. PARP-1 is also shown to be involved in necroptosis by at least two mechanisms. First, PARP-1 consumes both NAD^+^ and ATP, and NAD^+^ depletion triggers macrophage necroptosis under *Mycobacterium tuberculosis* infection [80]. Secondly, RIP1 can be modified by PARylation to trigger necroptosis [53]. Supporting this notion, we observed that PARP-1 inhibition blocks LPS-induced MLKL activation in both control and shNLRX1 cells. Nevertheless, the increased MLKL activation in shNLRX1 cells was still observed upon olaparib treatment for 6 h. Therefore, we suggest that NLRX1 can negatively regulate LPS-induced necroptosis through attenuating MLKL phosphorylation via a PARP-1-independent mechanism.

In summary, LPS induces a mixed type of cell death in microglia, which is protected by HO-1 induction and PARP-1 activation but involves necroptosis. NLRX1 exerts multifaceted actions in these death-regulating pathways and causes an integrated cell protection. NLRX1 upregulates LPS-induced HO-1 expression via the positive loop between Nrf2 and p62. NLRX1 positively regulates PARP-1 activation and DNA repair via ROS- and autophagy-independent pathways. NLRX1 also attenuates necroptosis by reducing MLKL phosphorylation independent of PARP-1.

## Data Availability

All data generated or analyzed during this study are included in this published article (and Supplementary Materials).

## Supplementary Materials

The following supporting information can be downloaded at https://www.mdpi.com/article/10.3390/antiox13040481/s1, Table S1: Table of qPCR primers.

## Abbreviations

DAPI	4′,6-diamidino-2-phenylindole
DCFDA	Dichlorodihydrofluorescein diacetate
DMEM	Dulbecco’s modified Eagle’s medium
FBS	Fetal bovine serum
H_2_O_2_	Hydrogen peroxide
HO-1	Heme oxygenase-1
IFN	Interferon
LC3	Microtubule-associated protein 1A/1B-light chain 3
LPS	Lipopolysaccharide
MAVS	Mitochondrial antiviral-signaling protein
MLKL	Mixed lineage kinase domain-like pseudokinase
NAC	N-acetyl-L-cysteine
NLR	NOD-like receptor
NLRX1	Nucleotide-binding oligomerization domain, leucine-rich repeat-containing X1
PAR	Poly (ADP-ribose)
PARP-1	Poly (ADP-ribose) polymerase-1
PBS	Phosphate buffered saline
PI	Propidium iodide
RIPK1	Receptor-interacting serine/threonine-protein kinase 1
ROS	Reactive oxygen species
RT-PCR	Reverse-transcription and real-time polymerase chain reaction
SDS	Sodium dodecyl sulfate
TBST	Tris-buffer saline with Tween 20
ZnPP	Zinc protoporphyrin
