# Supplementary material for: NLRX1 Inhibits LPS-Induced Microglial Death via Inducing p62-Dependent HO-1 Expression, Inhibiting MLKL and Activating PARP-1
Source: Antioxidants (Basel). 2024 Apr 17;13(4):481. doi: 10.3390/antiox13040481 (PMC11047433; doi:10.3390/antiox13040481)
Supplement: Supplementary file 1 [file antioxidants-13-00481-s001.zip › antioxidants-2934227-supplementary.pdf]

Table S1. qPCR primers.

Gene	Gene Accession Number	Forward (5′ to 3′)	Reverse (3′ to 5′)
**β-actin**	NM_007393	GAC TAC CTC ATG AAG AT C CT	CCA CAT CTG CTG GAA GGT GG
**NLRX1**	NM_001163743	CCT CTG CTC TTC AAC TT G CTC	CCC ATC TGA TCC AGA ACA TCG
**iNOS**	NM_001313922	CAG CTG GGC TGT ACA A AC CTT	CAT TGG AAG TGA AGC GTT TCG
**TNF-α**	NM_013693.3	ATG AGA AGT TCC CAA AT G GCC	TCC ACT TGG TGG TTT G CT ACG
**Keap1**	NM_001110307.1	GAT CTA CGT CCT CGG AG G CT	TCA CTG TCC GGG TCA T AG CA
**HO-1**	NM_010442.2	CAG AGC CGT CTC GAG C AT AG	CAA ATC CTG GGG CAT GCT GT
**Nrf2**	NM_010902.5	CCA CAT TCC CAA ACA AG A TGC C	ATC CAG GGC AAG CGA CTC AT
**p62**	NM_001290769.1	CCT CAG CCC TCT AGG CA T TG	TTC TGG GGT AGT GGG T GT CA
**LC3**	NM_025735.3	GTC ACC CAG GCG AGT TA C C	TTA CAG CGG TCG GCG AAG
**MLKL**	XM_036154342.1	TCT TTC TGG CAG AGA AC G AAT CT	TCT TAC ACC TTC TTG T CC GTG G
